# Supplementary material for: Effect of the Trunk and Upper Limb Passive Stabilization on Hand Movements and Grip Strength Following Various Types of Strokes—An Observational Cohort Study
Source: Brain Sci. 2022 Sep 13;12(9):1234. doi: 10.3390/brainsci12091234 (PMC9497157; doi:10.3390/brainsci12091234)
Supplement: Supplementary file 1 [file brainsci-12-01234-s001.zip › brainsci-1898368-supplementary.pdf]

Table S1. Descriptive statistics for patients after a cerebrum hemorrhagic stroke in Groups I and II.

	N	M	Me	SD	Sk.	Kurt.	Min.	Maks.
Group I RM [mm]								
Sitting position								
Wrist active movement	10	20.50	20.00	6.50	-0.36	0.26	8	30
Wrist passive movement	10	25.50	26.00	4.97	-0.27	0.76	16	34
F5 active	10	19.70	19.50	8.72	-0.01	0.37	4	35
F5 passive	10	23.80	21.00	8.38	0.77	-0.08	13	40
F4 active	10	23.40	25.00	7.01	-1.43	2.93	7	32
F4 passive	10	28.70	28.50	4.55	-0.84	1.28	19	35
F3 active	10	23.50	25.50	6.88	-1.16	0.84	9	31
F3 passive	10	28.10	28.50	3.87	-1.42	3.06	19	33
F2 active	10	21.70	24.00	7.04	-2.45	6.70	3	27
F2 passive	10	28.20	28.50	2.97	-0.97	0.90	22	32
F1 active	10	12.80	14.00	5.25	-1.24	2.29	1	20
F1 passive	10	16.90	18.00	3.99	-1.23	2.13	8	22
Lying with stabilization UL								
Wrist active movement	10	18.90	21.00	6.39	-1.24	1.19	5	25
Wrist passive movement	10	25.60	25.50	4.01	-0.35	0.32	18	32
F5 active	10	18.20	17.00	8.51	0.35	0.55	4	34
F5 passive	10	22.00	18.00	8.29	0.17	-1.28	9	34
F4 active	10	22.60	25.00	9.45	-0.32	-1.62	9	34
F4 passive	10	26.50	28.00	8.21	-0.39	-1.41	14	36
F3 active	10	23.10	25.00	5.97	-0.55	-1.33	13	29
F3 passive	10	27.30	30.00	6.48	-0.60	-1.17	16	34
F2 active	10	20.50	20.50	6.19	-0.50	-0.81	10	28
F2 passive	10	26.20	24.50	3.91	0.55	-1.67	22	32
F1 active	10	12.50	13.00	4.77	-0.31	-0.96	5	19
F1 passive	10	17.80	18.00	3.49	-0.50	0.23	11	23
Group II RM [mm]								
Lying without stabilization UL								
Wrist active movement	10	17.80	17.50	2.74	-0.27	-0.78	13	21
Wrist passive movement	10	24.50	24.50	2.64	0.87	0.58	22	30
F5 active	10	17.10	18.50	7.19	0.15	-0.47	8	30
F5 passive	10	20.20	19.50	6.27	0.48	0.06	11	32
F4 active	10	21.60	22.00	7.28	0.68	1.50	11	37
F4 passive	10	24.00	25.00	6.36	0.37	1.63	13	37
F3 active	10	22.10	22.50	4.51	-0.36	-1.27	15	28
F3 passive	10	24.20	25.00	5.61	-0.20	-1.00	15	32
F2 active	10	19.70	19.50	3.89	0.35	-0.94	15	26
F2 passive	10	24.00	24.00	3.83	0.09	0.18	18	31
F1 active	10	6.10	5.50	2.85	-0.02	-0.24	1	10
F1 passive	10	8.20	8.00	1.81	-0.09	-0.43	5	11
Lying with stabilization UL								
Wrist active movement	10	17.80	17.50	2.74	-0.27	-0.78	13	21
Wrist passive movement	10	24.50	24.50	2.64	0.87	0.58	22	30
F5 active	10	17.10	18.50	7.19	0.15	-0.47	8	30

F5 passive	10	20.20	19.50	6.27	0.48	0.06	11	32
F4 active	10	21.60	22.00	7.28	0.68	1.50	11	37
F4 passive	10	24.00	25.00	6.36	0.37	1.63	13	37
F3 active	10	22.10	22.50	4.51	-0.36	-1.27	15	28
F3 passive	10	24.20	25.00	5.61	-0.20	-1.00	15	32
F2 active	10	19.70	19.50	3.89	0.35	-0.94	15	26
F2 passive	10	24.00	24.00	3.83	0.09	0.18	18	31
F1 active	10	6.10	5.50	2.85	-0.02	-0.24	1	10
F1 passive	10	8.20	8.00	1.81	-0.09	-0.43	5	11

Legend: M - mean; Me—median; SD - standard deviation; Sk. – skew; Kurt. - kurtosis; RM - Range of motion; F -finger; UL - upper limb.

Table S2. Descriptive statistics for patients after a cerebrum ischemic stroke in Groups I and II.

	N	M	Me	SD	Sk.	Kurt.	Min.	Maks.
Group I RM [mm]								
Sitting position								
Wrist active movement	40	16.85	16.50	5.49	0.10	-1.10	8	28
Wrist passive movement	40	24.15	24.00	3.79	0.08	0.44	15	34
F5 active	40	18.88	19.00	8.22	0.19	-0.19	4	38
F5 passive	40	21.75	21.50	7.68	0.47	0.33	7	41
F4 active	40	22.75	22.50	7.30	-0.07	-0.89	9	35
F4 passive	40	25.90	25.50	6.71	-0.01	-0.52	13	40
F3 active	40	22.45	23.00	5.56	-0.20	-0.95	11	32
F3 passive	40	25.35	25.50	5.96	-0.39	-0.02	11	35
F2 active	40	19.13	19.50	5.32	0.03	-0.71	8	29
F2 passive	40	23.38	24.00	5.39	-0.06	-0.45	12	34
F1 active	40	8.08	7.00	5.42	0.93	0.56	1	24
F1 passive	40	12.45	11.00	6.43	0.83	0.70	2	30
Lying with stabilization UL								
Wrist active movement	40	19.58	21.00	5.06	-0.91	1.26	4	28
Wrist passive movement	40	26.20	27.00	3.77	-0.80	0.83	16	33
F5 active	40	17.08	17.00	6.91	-0.05	-0.25	1	31
F5passive	40	20.70	20.00	6.39	0.21	-0.35	7	35
F4 active	40	21.53	22.50	6.31	-0.36	-0.37	7	32
F4 passive	40	25.08	24.50	6.01	0.48	0.72	14	43
F3 active	40	22.20	23.00	5.53	-0.37	-0.67	11	32
F3 passive	40	25.45	26.00	5.09	-0.18	0.70	12	38
F2 active	40	20.60	21.00	5.24	-0.66	0.58	5	29
F2 passive	40	24.30	25.00	4.91	-0.39	0.26	13	36
F1 active	40	7.75	7.00	4.29	1.13	1.22	1	21
F1 passive	40	11.98	11.00	5.47	1.36	1.48	5	28
Group II RM [mm]								
Lying without stabilization UL								
Wrist active movement	40	17.53	18.00	4.63	-0.19	-0.85	8	27
Wrist passive movement	40	25.78	26.00	4.74	0.09	-0.06	16	38
F5 active	40	16.72	17.00	6.68	-0.02	-0.77	4	29
F5 passive	40	19.50	20.50	6.17	-0.37	-0.53	7	30
F4 active	40	20.82	22.00	6.71	-0.37	-0.33	4	34
F4 passive	40	23.58	24.00	5.20	-0.22	-0.14	12	35

F3 active	40	20.73	21.50	5.50	-0.80	0.63	6	30
F3 passive	40	23.77	23.50	4.42	-0.10	-0.31	14	34
F2 active	40	17.78	19.00	5.03	-0.18	0.52	5	32
F2 passive	40	21.62	22.00	4.27	0.43	1.05	11	34
F1 active	40	5.73	5.00	3.15	0.60	-0.20	1	13
F1 passive	40	7.75	7.00	3.49	0.89	0.44	2	18
Lying with stabilization UL								
Wrist active movement	40	17.53	18.00	4.63	-0.19	-0.85	8	27
ZR nadg passive movement	40	25.78	26.00	4.74	0.09	-0.06	16	38
F5 active	40	16.72	17.00	6.68	-0.02	-0.77	4	29
F5 passive	40	19.50	20.50	6.17	-0.37	-0.53	7	30
F4 active	40	20.82	22.00	6.71	-0.37	-0.33	4	34
F4 passive	40	23.58	24.00	5.20	-0.22	-0.14	12	35
F3 active	40	20.73	21.50	5.50	-0.80	0.63	6	30
F3 passive	40	23.77	23.50	4.42	-0.10	-0.31	14	34
F2 active	40	17.78	19.00	5.03	-0.18	0.52	5	32
F2 passive	40	21.62	22.00	4.27	0.43	1.05	11	34
F1 active	40	5.73	5.00	3.15	0.60	-0.20	1	13
F1 passive	40	7.75	7.00	3.49	0.89	0.44	2	18

Legend: M - mean; Me—median; SD - standard deviation; Sk. – skew; Kurt. - kurtosis; RM - Range of motion; F -finger; UL - upper limb.

Table S3. Descriptive statistics after a cerebellar hemorrhagic stroke in Groups I and II.

	N	M	Me	SD	Sk.	Kurt.	Min.	Maks.
Group I RM [mm]								
Sitting position								
Wrist active movement	5	18.20	19.00	2.59	-0.36	-2.41	15	21
Wrist passive movement	5	24.00	25.00	2.35	-0.58	-2.63	21	26
F5 active	5	16.20	16.00	4.21	-0.60	0.27	10	21
F5 passive	5	21.00	22.00	4.00	-1.99	4.21	14	24
F4 active	5	20.20	22.00	4.55	-0.59	-1.63	14	25
F4 passive	5	26.60	26.00	5.32	0.21	-2.56	21	33
F3 active	5	21.60	21.00	3.85	0.07	-2.28	17	26
F3 passive	5	26.40	25.00	5.37	0.38	-2.61	21	33
F2 active	5	20.20	21.00	2.17	-0.91	-0.74	17	22
F2 passive	5	29.60	29.00	9.29	1.00	0.70	21	44
F1 active	5	7.00	6.00	3.32	0.41	-2.84	4	11
F1 passive	5	8.20	10.00	3.42	-0.60	-2.98	4	11
Lying with stabilization UL								
Wrist active movement	5	16.40	18.00	3.78	-0.79	-1.25	11	20
Wrist passive movement	5	20.40	21.00	2.07	-1.45	1.93	17	22
F5 active	5	12.20	13.00	4.66	-0.43	-1.84	6	17
F5 passive	5	13.00	13.00	4.53	0.00	-2.76	8	18
F4 active	5	16.60	16.00	5.41	0.98	0.93	11	25
F4 passive	5	18.40	17.00	3.78	1.98	4.03	16	25
F3 active	5	18.80	20.00	5.63	0.58	-0.30	13	27
F3 passive	5	20.80	21.00	4.21	0.60	0.27	16	27
F2 active	5	19.80	18.00	4.27	1.65	3.01	16	27

F2 passive	5	21.80	22.00	3.35	0.89	1.47	18	27
F1 active	5	7.40	7.00	1.52	1.75	3.72	6	10
F1 passive	5	9.60	9.00	2.70	1.34	2.02	7	14
Group II RM [mm]								
Lying without stabilization UL								
Wrist active movement	5	18.60	18.00	1.52	1.12	1.46	17	21
Wrist passive movement	5	23.00	23.00	2.83	0.00	2.00	19	27
F5 active	5	17.00	17.00	1.23	-1.36	2.00	15	18
F5 passive	5	20.60	18.00	6.39	2.21	4.91	17	32
F4 active	5	18.20	17.00	3.70	0.38	-1.81	14	23
F4 passive	5	19.20	20.00	3.11	-0.06	-2.30	16	23
F3 active	5	20.80	22.00	4.44	-1.01	0.27	14	25
F3 passive	5	21.40	24.00	4.28	-1.03	-0.65	15	25
F2 active	5	15.40	17.00	6.03	-1.86	3.69	5	20
F2 passive	5	19.40	19.00	2.51	0.20	1.50	16	23
F1 active	5	7.00	7.00	2.55	0.91	2.00	4	11
F1 passive	5	7.40	7.00	2.30	1.03	1.13	5	11
Lying with stabilization UL								
Wrist active movement	5	18.60	18.00	1.52	1.12	1.46	17	21
Wrist passive movement	5	23.00	23.00	2.83	0.00	2.00	19	27
F5 active	5	17.00	17.00	1.23	-1.36	2.00	15	18
F5 passive	5	20.60	18.00	6.39	2.21	4.91	17	32
F4 active	5	18.20	17.00	3.70	0.38	-1.81	14	23
F4 passive	5	19.20	20.00	3.11	-0.06	-2.30	16	23
F3 active	5	20.80	22.00	4.44	-1.01	0.27	14	25
F3 passive	5	21.40	24.00	4.28	-1.03	-0.65	15	25
F2 active	5	15.40	17.00	6.03	-1.86	3.69	5	20
F2 passive	5	19.40	19.00	2.51	0.20	1.50	16	23
F1 active	5	7.00	7.00	2.55	0.91	2.00	4	11
F1 passive	5	7.40	7.00	2.30	1.03	1.13	5	11

Legend: M - mean; Me—median; SD - standard deviation; Sk. – skew; Kurt. - kurtosis; RM - Range of motion; F -finger; UL - upper limb.

Table S4. Descriptive statistics for patients after cerebellar ischemic stroke in Groups I and II.

	N	M	Me	SD	Sk.	Kurt.	Min.	Maks.
Group I RM [mm]								
Sitting position								
Wrist active movement	5	17.20	19.00	7.73	-1.74	3.48	4	24
Wrist passive movement	5	26.00	26.00	3.81	0.00	-0.38	21	31
F5 active	5	24.40	29.00	8.96	-1.86	3.37	9	30
F5 passive	5	27.80	29.00	8.98	-0.70	1.99	14	39
F4 active	5	27.60	29.00	6.80	-1.69	3.56	16	34
F4 passive	5	30.00	31.00	6.60	-1.53	2.70	19	36
F3 active	5	25.20	29.00	5.72	-0.65	-2.90	18	30
F3 passive	5	28.60	30.00	3.98	-1.54	2.36	22	32
F2 active	5	23.20	23.00	3.03	-0.23	-0.14	19	27
F2 passive	5	28.20	28.00	2.39	-0.21	-1.12	25	31
F1 active	5	9.00	8.00	4.74	1.64	2.95	5	17

F1 passive	5	10.40	9.00	4.10	1.19	2.10	6	17
Lying with stabilization UL								
Wrist active movement	5	22.20	21.00	4.44	2.04	4.35	19	30
Wrist passive movement	5	26.60	26.00	3.29	1.43	2.09	24	32
F5 active	5	22.80	25.00	6.87	-1.78	3.74	11	29
F5 passive	5	23.60	25.00	5.64	-1.67	3.56	14	29
F4 active	5	27.00	27.00	4.95	0.00	2.00	20	34
F4 passive	5	28.80	27.00	3.03	1.84	3.26	27	34
F3 active	5	26.20	28.00	3.90	-0.46	-3.12	22	30
F3 passive	5	29.80	30.00	1.79	0.05	-2.32	28	32
F2 active	5	22.40	23.00	3.05	-0.54	0.00	18	26
F2 passive	5	27.40	27.00	1.14	0.41	-0.18	26	29
F1 active	5	6.40	6.00	2.97	-0.55	0.87	2	10
F1 passive	5	7.80	7.00	1.64	0.52	-1.69	6	10
Group II RM [mm]								
Lying without stabilization UL								
Wrist active movement	5	16.40	15.00	3.91	0.29	-2.51	12	21
Wrist passive movement	5	24.20	24.00	1.48	-0.55	0.87	22	26
F5 active	5	13.40	14.00	4.28	-0.46	1.93	7	19
F5 passive	5	14.80	15.00	4.15	-1.34	2.45	8	19
F4 active	5	17.80	18.00	4.66	-0.31	1.61	11	24
F4 passive	5	19.60	18.00	5.03	1.55	2.70	15	28
F3 active	5	19.20	21.00	4.71	-1.93	4.05	11	23
F3 passive	5	21.40	22.00	5.81	-1.14	2.63	12	28
F2 active	5	15.80	16.00	2.86	-0.31	-1.54	12	19
F2 passive	5	19.40	19.00	4.04	0.12	0.68	14	25
F1 active	5	4.20	3.00	2.17	0.56	-2.37	2	7
F1 passive	5	6.60	7.00	2.07	-0.24	-1.96	4	9
Lying with stabilization UL								
Wrist active movement	5	16.40	15.00	3.91	0.29	-2.51	12	21
Wrist passive movement	5	24.20	24.00	1.48	-0.55	0.87	22	26
F5 active	5	13.40	14.00	4.28	-0.46	1.93	7	19
F5 passive	5	14.80	15.00	4.15	-1.34	2.45	8	19
F4 active	5	17.80	18.00	4.66	-0.31	1.61	11	24
F4 passive	5	19.60	18.00	5.03	1.55	2.70	15	28
F3 active	5	19.20	21.00	4.71	-1.93	4.05	11	23
F3 passive	5	21.40	22.00	5.81	-1.14	2.63	12	28
F2 active	5	15.80	16.00	2.86	-0.31	-1.54	12	19
F2 passive	5	19.40	19.00	4.04	0.12	0.68	14	25
F1 active	5	4.20	3.00	2.17	0.56	-2.37	2	7
F1 passive	5	6.60	7.00	2.07	-0.24	-1.96	4	9

Legend: M - mean; Me—median; SD - standard deviation; Sk. – skew; Kurt. - kurtosis; RM - Range of motion; F -finger; UL - upper limb.

Table S5. Comparison of patients with different types of stroke in terms of their range of motion, frequency of movement, and handgrip strength in sitting and lying positions with the upper limb against the patient's body in Group I.

	Group I	N	Average Rank	M	Me	SD	H	p	η^2
Sitting position									
Hz Wrist [cyc/sec]	Cerebrum hemorrhagic stroke	10	33.15	1.25	1.05	0.87	2.61	0.456	<0.01
	Cerebrum ischemic stroke	40	28.58	1.05	0.80	0.65			
	Cerebellum hemorrhagic stroke	5	41.20	1.64	1.50	0.84			
	Cerebellum ischemic stroke	5	29.90	1.20	0.70	0.87			
MaxROM Wrist [mm]	Cerebrum hemorrhagic stroke	10	39.35	21.46	19.45	1.,60	12.20	**0.007**	0.08
	Cerebrum ischemic stroke	40	25.06	14.03	14.00	5.37			
	Cerebellum hemorrhagic stroke	5	40.40	19.10	19.20	1.01			
	Cerebellum ischemic stroke	5	46.40	20.72	21.70	4.18			
HzF5	Cerebrum hemorrhagic stroke	10	37.75	1.87	1.95	0.85	2.58	0.461	<0.01
	Cerebrum ischemic stroke	40	28.94	1.45	1.15	0.93			
	Cerebellum hemorrhagic stroke	5	33.40	1.54	1.30	0.72			
	Cerebellum ischemic stroke	5	25.60	1.28	0.80	0.90			
MaxROM	Cerebrum hemorrhagic stroke	10	29.65	17.25	18.20	9.18	2.60	0.458	<0.01
	Cerebrum ischemic stroke	40	29.23	17.36	16.65	8.01			
	Cerebellum hemorrhagic stroke	5	30.40	16.80	21.20	9.79			
	Cerebellum ischemic stroke	5	42.50	22.90	25.30	6.84			
HzF4	Cerebrum hemorrhagic stroke	10	38.15	1.86	1.95	0.84	2.80	0.424	<0.01
	Cerebrum ischemic stroke	40	28.78	1.43	1.15	0.95			
	Cerebellum hemorrhagic stroke	5	33.50	1.54	1.30	0.72			
	Cerebellum ischemic stroke	5	26.00	1.28	0.80	0.90			
MaxROM	Cerebrum hemorrhagic stroke	10	32.90	22.13	22.90	9.08	4.69	0.196	0.01
	Cerebrum ischemic stroke	40	28.11	21.26	21.10	7.81			
	Cerebellum hemorrhagic stroke	5	29.70	21.14	21.80	6.14			
	Cerebellum ischemic stroke	5	45.60	27.64	26.60	4.64			
HzF3	Cerebrum hemorrhagic stroke	10	38.25	1.85	2.00	0.88	2.53	0.470	<0.01
	Cerebrum ischemic stroke	40	29.18	1.44	1.15	0.93			
	Cerebellum hemorrhagic stroke	5	30.00	1.42	1.00	0.81			
	Cerebellum ischemic stroke	5	26.10	1.28	0.80	0.90			
MaxROM	Cerebrum hemorrhagic stroke	10	38.25	22.43	23.95	7.51	5.69	0.128	0.02
	Cerebrum ischemic stroke	40	26.98	20.29	19.75	4.95			
	Cerebellum hemorrhagic stroke	5	32.00	21.52	22.50	4.97			
	Cerebellum ischemic stroke	5	41.70	24.86	25.30	4.54			
HzF2	Cerebrum hemorrhagic stroke	10	37.95	1.85	2.00	0.88	2.39	0.495	<0.01
	Cerebrum ischemic stroke	40	29.34	1.45	1.15	0.92			
	Cerebellum hemorrhagic stroke	5	29.70	1.42	1.00	0.81			
	Cerebellum ischemic stroke	5	25.70	1.28	0.80	0.90			
MaxROM	Cerebrum hemorrhagic stroke	10	34.10	18.22	20.00	6.79	6.45	0.091	0.03
	Cerebrum ischemic stroke	40	26.80	16.85	17.60	4.81			
	Cerebellum hemorrhagic stroke	5	39.80	20.60	20.10	6.00			
	Cerebellum ischemic stroke	5	43.60	21.48	21.90	3.20			
HzF1	Cerebrum hemorrhagic stroke	10	38.30	1.72	2.00	1.01	3.13	0.372	<0.01
	Cerebrum ischemic stroke	40	27.90	1.11	0.90	0.89			
	Cerebellum hemorrhagic stroke	5	33.80	1.38	1.00	0.94			

	Cerebellum ischemic stroke	5	32.40	1.28	0.80	0.90			
MaxROM	Cerebrum hemorrhagic stroke	10	39.30	11.67	12.10	5.23	4.21	0.240	0.01
	Cerebrum ischemic stroke	40	27.41	8.13	7.25	5.34			
	Cerebellum hemorrhagic stroke	5	33.60	11.20	10.00	8.87			
	Cerebellum ischemic stroke	5	34.50	10.66	9.60	5.64			
Grip strength [kg]	Cerebrum hemorrhagic stroke	10	29.60	19.82	14.95	17.02	1.65	0.647	<0.01
	Cerebrum ischemic stroke	40	29.11	17.25	15.05	11.38			
	Cerebellum hemorrhagic stroke	5	36.40	25.32	22.70	18.96			
	Cerebellum ischemic stroke	5	37.50	23.28	18.40	12.64			
Lying with stabilization UL									
Hz Wrist [cyc/sec]	Cerebrum hemorrhagic stroke	10	38.70	1.56	1.50	1.15	3.41	0.333	<0.01
	Cerebrum ischemic stroke	40	28.06	1.00	0.85	0.65			
	Cerebellum hemorrhagic stroke	5	35.20	1.32	1.20	0.90			
	Cerebellum ischemic stroke	5	28.90	1.02	0.70	0.72			
MaxROM [mm]	Cerebrum hemorrhagic stroke	10	30.85	22.58	20.25	14.20	1.76	0.623	<0.01
	Cerebrum ischemic stroke	40	29.23	19.06	21.10	6.37			
	Cerebellum hemorrhagic stroke	5	30.30	20.60	21.00	2.65			
	Cerebellum ischemic stroke	5	40.20	23.60	22.00	4.61			
HzF5	Cerebrum hemorrhagic stroke	10	39.95	2.05	1.85	0.71	4.58	0.206	0.01
	Cerebrum ischemic stroke	40	27.94	1.54	1.25	1.00			
	Cerebellum hemorrhagic stroke	5	36.10	1.84	1.50	0.79			
	Cerebellum ischemic stroke	5	26.50	1.46	0.90	1.10			
MaxROM	Cerebrum hemorrhagic stroke	10	32.10	19.78	19.05	14.61	3.20	0.362	<0.01
	Cerebrum ischemic stroke	40	28.84	16.20	15.60	7.79			
	Cerebellum hemorrhagic stroke	5	27.90	15.24	18.20	7.52			
	Cerebellum ischemic stroke	5	43.20	22.02	23.00	7.87			
HzF4	Cerebrum hemorrhagic stroke	10	41.05	2.08	1.85	0.74	5.74	0.125	0.02
	Cerebrum ischemic stroke	40	27.60	1.49	1.15	1.01			
	Cerebellum hemorrhagic stroke	5	36.90	1.86	1.50	0.83			
	Cerebellum ischemic stroke	5	26.20	1.44	0.80	1.11			
MaxROM	Cerebrum hemorrhagic stroke	10	36.30	21.47	22.40	7.35	4.57	0.206	0.01
	Cerebrum ischemic stroke	40	27.46	1.49	1.15	1.01			
	Cerebellum hemorrhagic stroke	5	31.50	21.04	18.60	9.57			
	Cerebellum ischemic stroke	5	42.20	23.62	25.50	4.47			
HzF3	Cerebrum hemorrhagic stroke	10	40.30	2.08	1.85	0.74	4.91	0.178	0.02
	Cerebrum ischemic stroke	40	27.96	1.54	1.25	1.00			
	Cerebellum hemorrhagic stroke	5	36.10	1.84	1.50	0.79			
	Cerebellum ischemic stroke	5	25.60	1.44	0.80	1.11			
MaxROM	Cerebrum hemorrhagic stroke	10	33.45	20.58	21.30	5.34	2.00	0.573	<0.01
	Cerebrum ischemic stroke	40	28.35	19.19	19.50	5.04			
	Cerebellum hemorrhagic stroke	5	34.80	21.24	20.20	6.33			
	Cerebellum ischemic stroke	5	37.50	22.08	20.00	3.58			
HzF2	Cerebrum hemorrhagic stroke	10	40.30	2.08	1.85	0.74	4.94	0.176	0.02
	Cerebrum ischemic stroke	40	27.95	1.54	1.25	1.00			
	Cerebellum hemorrhagic stroke	5	36.20	1.86	1.50	0.83			
	Cerebellum ischemic stroke	5	25.60	1.44	0.80	1.11			
MaxROM	Cerebrum hemorrhagic stroke	10	30.95	16.85	17.85	5.44	4.04	0.258	0.01
	Cerebrum ischemic stroke	40	27.94	16.50	15.90	4.80			
	Cerebellum hemorrhagic stroke	5	39.60	20.28	19.60	5.62			

	Cerebellum ischemic stroke	5	41.00	20.08	19.20	3.51			
HzF1	Cerebrum hemorrhagic stroke	10	37.55	1.64	1.80	1.15	2.78	0.427	<0.01
	Cerebrum ischemic stroke	40	27.99	1.04	0.90	0.78			
	Cerebellum hemorrhagic stroke	5	32.80	1.42	1.20	1.27			
	Cerebellum ischemic stroke	5	34.20	1.44	0.80	1.11			
MaxROM	Cerebrum hemorrhagic stroke	10	34.25	9.01	10.15	4.47	3.11	0.375	<0.01
	Cerebrum ischemic stroke	40	28.04	7.48	7.15	4.86			
	Cerebellum hemorrhagic stroke	5	41.00	11.94	13.50	6.59			
	Cerebellum ischemic stroke	5	32.20	8.34	9.20	3.85			
Grip strength [kg]	Cerebrum hemorrhagic stroke	10	29.30	20.38	13.95	17.14	1.29	0.731	<0.01
	Cerebrum ischemic stroke	40	29.44	18.13	15.55	11.53			
	Cerebellum hemorrhagic stroke	5	37.90	26.20	22.70	17.47			
	Cerebellum ischemic stroke	5	34.00	21.44	16.70	13.66			

Legend: M – mean; Me – median; SD – standard deviation; H - Kruskal Wallis test; p - test probability; η^2 - wielkość efektu; ROM – range of motion from flexion to extension; one cycle = the movement from flexion to extension.

Table S6. Comparison of patients with different types of stroke in terms of their range of motion, frequency of movement, and handgrip strength in sitting and lying positions with the upper limb against the patient's body in Group II.

	Group II	N	Average Rank	M	Me	SD	H	p	η^2
Lying without stabilization UL									
Hz Wrist [cyc/sec]	Cerebrum hemorrhagic stroke	10	21.20	0.77	0.70	0.49	9.13	**0.028**	0.05
	Cerebrum ischemic stroke	40	43.22	1.53	1.45	0.90			
	Cerebellum hemorrhagic stroke	5	51.60	1.78	1.90	0.91			
	Cerebellum ischemic stroke	5	35.40	1.20	1.20	0.56			
MaxROM nadg[mm]	Cerebrum hemorrhagic stroke	10	45.20	18.42	19.60	3.88	0.84	0.839	<0.01
	Cerebrum ischemic stroke	40	39.85	17.28	18.00	8.12			
	Cerebellum hemorrhagic stroke	5	44.20	18.50	18.50	2.60			
	Cerebellum ischemic stroke	5	35.20	16.20	15.80	4.90			
HzF5	Cerebrum hemorrhagic stroke	10	31.65	2.60	1.00	0.98	2.49	0.476	<0.01
	Cerebrum ischemic stroke	40	42.38	1.75	1.80	1.03			
	Cerebellum hemorrhagic stroke	5	43.50	1.72	1.50	0.71			
	Cerebellum ischemic stroke	5	32.70	1.26	1.30	0.47			
MaxROM	Cerebrum hemorrhagic stroke	10	46.25	16.45	18.20	6.82	3.96	0.265	0.01
	Cerebrum ischemic stroke	40	37.98	14.67	15.30	6.61			
	Cerebellum hemorrhagic stroke	5	57.30	19.66	20.10	4.17			
	Cerebellum ischemic stroke	5	42.40	15.94	16.10	6.57			
HzF4	Cerebrum hemorrhagic stroke	10	30.65	1.29	1.00	0.99	3.23	0.358	<0.01
	Cerebrum ischemic stroke	40	42.77	1.80	1.85	1.03			
	Cerebellum hemorrhagic stroke	5	42.40	1.72	1.50	0.71			
	Cerebellum ischemic stroke	5	31.10	1.24	1.20	0.59			
MaxROM	Cerebrum hemorrhagic stroke	10	39.30	19.24	17.75	5.71	1.73	0.629	<0.01
	Cerebrum ischemic stroke	40	39.89	18.48	18.70	9.31			

	Cerebellum hemorrhagic stroke	5	53.50	22.20	21.80	4.99			
	Cerebellum ischemic stroke	5	37.20	17.94	16.80	5.88			
HzF3	Cerebrum hemorrhagic stroke	10	30.25	1.29	1.00	0.99	3.93	0.269	0.01
	Cerebrum ischemic stroke	40	43.00	1.81	1.85	1.01			
	Cerebellum hemorrhagic stroke	5	42.60	1.72	1.50	0.71			
	Cerebellum ischemic stroke	5	28.90	1.18	1.20	0.50			
MaxROM	Cerebrum hemorrhagic stroke	10	47.40	20.29	21.15	3.51	4.03	0.259	0.01
	Cerebrum ischemic stroke	40	38.03	17.88	18.25	6.16			
	Cerebellum hemorrhagic stroke	5	56.80	22.08	21.90	3.72			
	Cerebellum ischemic stroke	5	40.10	18.34	18.40	4.82			
HzF2	Cerebrum hemorrhagic stroke	10	30.85	1.30	1.00	0.98	3.03	0.387	<0.01
	Cerebrum ischemic stroke	40	42.69	1.79	1.85	1.03			
	Cerebellum hemorrhagic stroke	5	42.40	1.72	1.50	0.71			
	Cerebellum ischemic stroke	5	31.60	1.26	1.30	0.47			
MaxROM	Cerebrum hemorrhagic stroke	10	46.80	16.50	16.85	3.60	2.74	0.434	<0.01
	Cerebrum ischemic stroke	40	38.53	14.79	15.65	6.79			
	Cerebellum hemorrhagic stroke	5	53.40	17.66	17.90	1.64			
	Cerebellum ischemic stroke	5	38.60	15.10	14.80	4.17			
HzF1	Cerebrum hemorrhagic stroke	10	43.05	1.29	1.00	0.98	1.28	0.734	<0.01
	Cerebrum ischemic stroke	40	40.26	1.24	1.00	1.04			
	Cerebellum hemorrhagic stroke	5	47.20	1.48	1.50	1.01			
	Cerebellum ischemic stroke	5	31.60	0.74	0.50	0.51			
MaxROM	Cerebrum hemorrhagic stroke	10	35.60	6.88	5.95	5.88	1.43	0.699	<0.01
	Cerebrum ischemic stroke	40	40.45	6.72	6.35	4.00			
	Cerebellum hemorrhagic stroke	5	50.80	8.24	7.40	2.25			
	Cerebellum ischemic stroke	5	40.60	6.78	4.60	3.71			
Grip strength [kg]	Cerebrum hemorrhagic stroke	10	41.45	19.86	17.15	12.65	0.61	0.895	<0.01
	Cerebrum ischemic stroke	40	41.23	20.00	19.15	12.48			
	Cerebellum hemorrhagic stroke	5	34.90	16.02	19.10	6.45			
	Cerebellum ischemic stroke	5	35.40	15.84	14.50	7.82			
Lying with stabilization UL									
Hz Wrist [cyc/sec]	Cerebrum hemorrhagic stroke	10	28.70	1.31	1.30	0.75	3.74	0.291	0.01
	Cerebrum ischemic stroke	40	42.03	1.84	1.60	0.99			
	Cerebellum hemorrhagic stroke	5	49.50	1.94	2.20	0.67			
	Cerebellum ischemic stroke	5	36.70	1.48	1.60	0.69			
MaxROM [mm]	Cerebrum hemorrhagic stroke	10	50.90	23.10	22.35	4.73	3.35	0.341	<0.01
	Cerebrum ischemic stroke	40	40.13	20.01	21.05	6.60			
	Cerebellum hemorrhagic stroke	5	30.20	17.80	17.00	3.67			
	Cerebellum ischemic stroke	5	34.40	19.28	16.00	6.71			
HzF5	Cerebrum hemorrhagic stroke	10	31.45	1.68	1.50	1.10	2.99	0.393	<0.01
	Cerebrum ischemic stroke	40	42.89	2.12	2.20	1.10			
	Cerebellum hemorrhagic stroke	5	39.30	1.98	1.80	0.58			
	Cerebellum ischemic stroke	5	31.10	1.58	1.90	0.72			
MaxROM	Cerebrum hemorrhagic stroke	10	41.50	15.64	16.30	5.38	4.06	0.255	0.01
	Cerebrum ischemic stroke	40	38.48	15.67	15.40	8.51			
	Cerebellum hemorrhagic stroke	5	60.00	20.12	19.50	3.07			
	Cerebellum ischemic stroke	5	43.20	15.60	18.70	8.00			
HzF4	Cerebrum hemorrhagic stroke	10	31.85	1.69	1.55	1.10	5.24	0.155	0.02

	Cerebrum ischemic stroke	40	43.46	2.16	2.15	1.04			
	Cerebellum hemorrhagic stroke	5	39.90	1.98	1.80	0.58			
	Cerebellum ischemic stroke	5	22.90	1.20	1.20	0.86			
MaxROM	Cerebrum hemorrhagic stroke	10	36.05	18.38	15.85	6.72	1.83	0.608	<0.01
	Cerebrum ischemic stroke	40	40.14	18.88	18.90	8.66			
	Cerebellum hemorrhagic stroke	5	53.00	22.42	19.90	5.65			
	Cerebellum ischemic stroke	5	41.20	18.82	20.00	8.94			
HzF3	Cerebrum hemorrhagic stroke	10	30.00	1.68	1.50	1.10	4.15	0.246	0.01
	Cerebrum ischemic stroke	40	43.33	2.21	2.20	1.02			
	Cerebellum hemorrhagic stroke	5	38.80	2.00	1.80	0.57			
	Cerebellum ischemic stroke	5	29.20	1.56	1.90	0.70			
MaxROM	Cerebrum hemorrhagic stroke	10	39.65	19.48	19.05	3.78	2.51	0.474	<0.01
	Cerebrum ischemic stroke	40	39.48	19.34	19.25	6.69			
	Cerebellum hemorrhagic stroke	5	56.40	24.44	26.20	6.51			
	Cerebellum ischemic stroke	5	38.50	18.40	20.80	7.18			
HzF2	Cerebrum hemorrhagic stroke	10	32.15	1.68	1.50	1.10	3.08	0.379	<0.01
	Cerebrum ischemic stroke	40	42.55	2.07	2.10	1.06			
	Cerebellum hemorrhagic stroke	5	44.00	2.10	1.90	0.52			
	Cerebellum ischemic stroke	5	29.10	1.48	1.60	0.72			
MaxROM	Cerebrum hemorrhagic stroke	10	42.15	1.68	1.50	1.10	3.78	0.286	0.01
	Cerebrum ischemic stroke	40	38.38	15.70	1.,80	5.62			
	Cerebellum hemorrhagic stroke	5	58.80	20.64	21.70	4.79			
	Cerebellum ischemic stroke	5	44.30	16.70	18.90	6.16			
HzF1	Cerebrum hemorrhagic stroke	10	30.65	0.82	0.70	0.81	7.91	**0.048**	0.04
	Cerebrum ischemic stroke	40	42.31	1.37	0.95	1.17			
	Cerebellum hemorrhagic stroke	5	57.00	2.00	1.80	0.57			
	Cerebellum ischemic stroke	5	22.00	0.32	0.40	0.16			
MaxROM	Cerebrum hemorrhagic stroke	10	32.80	8.22	5.95	8.19	2.48	0.479	<0.01
	Cerebrum ischemic stroke	40	40.38	8.22	8.35	4.34			
	Cerebellum hemorrhagic stroke	5	47.20	9.46	9.50	2.43			
	Cerebellum ischemic stroke	5	50.70	11.38	13.30	7.36			
Grip strength [kg]	Cerebrum hemorrhagic stroke	10	38.60	18.60	19.40	8.62	0.67	0.880	<0.01
	Cerebrum ischemic stroke	40	41.63	20.59	19.10	11.33			
	Cerebellum hemorrhagic stroke	5	36.60	17.14	19.70	5.59			
	Cerebellum ischemic stroke	5	34.6	17.04	17.3	7.05			

Legend: M – mean; Me – median; SD – standard deviation; H - Kruskal Wallis test; p - test probability; η^2 - wielkość efektu; ROM – range of motion from flexion to extension; one cycle = the movement from flexion to extension.
